# Taxonomic Profile of Cultivable Microbiota from Adult Sheep Follicular Fluid and Its Effects on In Vitro Development of Prepubertal Lamb Oocytes

**DOI:** 10.3390/ani15131951

**Published:** 2025-07-02

**Authors:** Slavcho Mrenoshki, Letizia Temerario, Antonella Mastrorocco, Grazia Visci, Elisabetta Notario, Marinella Marzano, Nicola Antonio Martino, Daniela Mrenoshki, Giovanni Michele Lacalandra, Graziano Pesole, Maria Elena Dell’Aquila

**Affiliations:** 1 Department of Microbiology and Immunology, Faculty of Veterinary Medicine, Ss. Cyril and Methodius University in Skopje, 1000 Skopje, North Macedonia; mrenoski@fvm.ukim.edu.mk; 2 Department of Biosciences, Biotechnology and Environment, University of Bari Aldo Moro, 70125 Bari, Italy; antonella.mastrorocco@uniba.it (A.M.); grazia.visci@uniba.it (G.V.); nicola.martino@uniba.it (N.A.M.); graziano.pesole@uniba.it (G.P.); mariaelena.dellaquila@uniba.it (M.E.D.); 3 Institute of Biomembranes, Bioenergetics and Molecular Biotechnologies, Consiglio Nazionale delle Ricerche, 70126 Bari, Italy; e.notario@ibiom.cnr.it (E.N.); m.marzano@ibiom.cnr.it (M.M.); 4 Department of Veterinary Medicine, University of Bari Aldo Moro, 70010 Valenzano, Italy; daniela.mrenoshki@uniba.it (D.M.); giovannimichele.lacalandra@uniba.it (G.M.L.)

**Keywords:** sheep, follicular fluid (FF), microbiota, bacterial cell-free supernatants (bCFSs), prepubertal lamb oocytes (PLOs), in vitro oocyte maturation (IVM), in vitro embryo culture (IVC)

## Abstract

The aims of the present study were to analyze the taxonomic profile and to evaluate the functional effects of sheep FF cultivable microbiota on prepubertal lamb oocytes PLOs developmental potential. Ovarian FFs were recovered from slaughtered adult sheep via the aspiration of developing follicles and used for microbiota propagation. Bacterial pellets underwent 16S rRNA gene sequencing and targeted culturomics, whereas cell-free supernatants were used as supplements for the in vitro maturation (IVM) of slaughtered PLOs. For the first time, bacteria presence in adult sheep FF was detected, with the first report of *Streptococcus infantarius* subsp. *infantarius* (as a species) and *Burkholderia cepacia* (as a genus and species) in either animal or human FF. The short- and long-term effects of bacterial metabolites on PLO maturation and embryonic development were demonstrated. As short-term effects, the addition of FF microbiota metabolites did not affect the oocyte nuclear maturation and mitochondria distribution pattern, except in one of the examined supernatants, which reduced all quantitative bioenergetic/oxidative parameters. As long-term effects, one of them reduced the total cleavage rate after in vitro embryo culture (IVC). In conclusion, microbiota/bacteria are present in adult sheep FF and may influence reproductive outcomes in vitro. Future studies may reveal the beneficial in vitro effects using the microbiome from preovulatory follicles.

## Simple Summary

Most of the studies conducted to date on the ovarian follicular fluid (FF) microbiome have been performed in humans with few studies in farm animals. Prepubertal lamb oocytes (PLOs) allow for applications in the juvenile in vitro embryo transfer (JIVET) technology and have translational relevance as models for human pediatric onco-fertility. However, despite extensive research effort, they still display lower developmental competence compared to their adult counterparts, due to the incomplete/perturbed nuclear/cytoplasmic maturation. Following the hypothesis that the FF of adult subjects could contain the microbiota metabolites essential for PLO acquisition of developmental competence, the aims of this study were to perform the taxonomic profiling of adult sheep FF cultivable bacteria, by combining 16s rRNA gene sequencing and (targeted) culturomics and to evaluate the functional effects of bacterial cell-free supernatants on PLO in vitro maturation and developmental potential. For the first time, we detected bacteria presence in adult sheep FF and demonstrated the variable short- and long-term effects of bacterial metabolites on PLO maturation and embryonic development.

## 1. Introduction

The majority of studies investigating microbiome metabolites effects on humans and animals have focused on the gut microbiota as it represents the most abundant, diverse, and functionally significant microbial community in the body. However, some bacteria colonize other animal body niches, including the female reproductive tract where plenty of evidence exists for the microbiota influence on reproductive health in mammals (reviewed in [1]). Still, current female reproductive tract microbiome studies related to the reproductive physiology and particularly infertility are primarily focused on the lower parts (predominantly vagina, but also cervix and uterus), while the scientific reports for the upper parts including ovaries and follicular fluid (FF) are rare and almost exclusively reserved for humans [2,3,4,5,6,7,8,9,10,11,12]. The majority of these reports provide microbiota detection in FFs collected from women undergoing trans-vaginal oocyte retrieval for in vitro fertilization (IVF), for which data are then coupled with IVF/pregnancy outcomes. Regarding domestic animals, to the best of our knowledge, there are only few studies on FF microbiota in pigs [13,14,15] and cattle [16], but not in sheep. Moreover, these studies lack evaluations of the microbiota influence on IVF and/or in vitro embryo culture (IVC) outcomes.

Prepubertal lamb oocytes (PLOs) are used for juvenile in vitro embryo transfer (JIVET) technology, an assisted reproductive technology (ART) that provides exciting opportunities in different fields of animal reproduction. Indeed, JIVET, together with artificial insemination (AI) and in vivo embryo production (or multiple ovulation and embryo transfer, MOET), allows for an increase in the genetic gain and reproductive efficiency while shortening the generation interval in breeding programs [17,18,19,20,21]. In addition, it fully falls in the logic of sustainable animal breeding and the preservation of bioresources, such as the germplasms of native or endangered animal breeds and species [22]. Moreover, in translational research for human reproductive medicine, PLOs represent unique models to study the events occurring in the oocyte between birth and puberty and to study the developmental potential of human oocytes recovered at pediatric and adolescent ages for their use in onco-fertility preservation strategies [23]. Despite their scientific and applied interest, the PLOs’ greatest limitation is their lower developmental competence in comparison to their adult counterparts [24,25,26]. This reduced competence has been related to incomplete or perturbed cytoplasmic, molecular, and nuclear maturation [27,28,29,30,31,32] but could be also due to differences in the composition of their FFs [33].

The follicular fluid (FF) is the biological microenvironment to which oocytes are naturally exposed during in vivo maturation. This complex and dynamic fluid comprises a myriad of stimuli from hormones, growth factors, lipids, antioxidants, and other metabolites arising through interactions with surrounding fluids and resident cumulus, granulosa, and theca cells [34]. Both early and recent studies have demonstrated that supplementing an in vitro maturation (IVM) medium with FF creates a more favorable environment for oocyte maturation and embryo development to the blastocyst stage compared to fetal calf serum (FCS) alone [34,35,36,37,38,39,40,41,42]. However, this topic is controversial, as other studies reported the inhibitory effects of FF addition in IVM due to variable FF contents in relation to the follicle size and reproductive cycle stage [43,44]. The positive effects of adult FF in the IVM medium of PLOs have been reported [40,45]. More recent studies, in humans and animals, have deepened the proteomic and metabolomic characterization of FF from the ovaries of adults [46,47,48,49,50,51] and prepubertal females [52,53,54]. The results of such studies allow us to identify factors responsible for the (positive or negative) effects of FF, as well as their origin. However, they did not investigate the microbiota presence in the FF and the possibility that some of the detected factors could be of microbial origin, produced locally in the FF, or remotely in the other body sites and then transported by the bloodstream to the ovary.

The aims of this study were to analyze the taxonomic profile and to evaluate the functional effects of the sheep FF cultivable microbiota on PLOs’ developmental potential. The first aim was achieved through the detection, isolation, and identification of the cultivable microbiota in the FF of adult sheep ovaries. The second one was realized by evaluating the effects of cultivable-microbiota-produced metabolites on PLO developmental competence by supplementing the IVM medium used in the JIVET procedure.

## 2. Materials and Methods

### 2.1. Collection and Transport of Ovaries

Sheep reproductive tracts were obtained at local slaughterhouses in the Puglia region, from commercially slaughtered healthy animals subjected to the routine veterinary inspection by the official veterinarian. Organs were transported, within 2–4 h after slaughter, at room temperature (25–30 °C), in a plastic container specialized for the short-distance transport of animal organs for in vitro studies.

#### 2.1.1. Cumulus–Oocyte Complex (COC) Retrieval

For cumulus–oocyte complex (COC) retrieval, ovaries from prepubertal lambs (less than 6 months of age) belonging to local and other national/international sheep breeds were used. Once arrived at the laboratory, the ovaries were processed by using the slicing procedure [55] to obtain COCs collected in a phosphate buffered saline (PBS) solution. For further culture, only those COCs with intact cumulus cell layers and homogeneous cytoplasm were used. In this study, 1175 COCs were recovered from the ovaries of 34 prepubertal lambs.

#### 2.1.2. Follicular Fluid (FF) Collection, Microbiota Propagation, and Supernatant Preparation

For FF collection, reproductive tracts from adult sheep were decontaminated by washing with sterile saline/PBS and subsequently processed in sterilized or disinfected aluminum containers. Decontamination was repeated on separated ovaries. Decontaminated ovaries were transferred to sterile petri dishes, and the FFs were collected via aspiration using a 1 ml insulin syringe with a 27 G, 0.4 × 12.7 mm needle (Insumed Syringes, Pic Solution, Casnate con Bernate, Italy). The aspirated FFs from 2–3 of the largest developing follicles, pooled for each ovary pair as one sample for further investigation, were collected and cultivated the same day or frozen in sterile cryovials at −80 °C for further use. For the cultivation of FFs, we used thioglycolate broth (Fluid Thioglycollate Medium, Liofilchem s.r.l., Roseto degli Abruzzi, Italy, ref. 610050; TgB). TgB was inoculated with at least 10 µL of FF, and tubes were incubated at 37 °C for 7 days in a normal atmosphere. One uninoculated tube of broth was also incubated in the same way and used as a negative control (negative broth control, negBRTH). In the same way, we also cultivated tubes with known and previously identified bacterial species of *Escherichia coli* and *Staphylococcus aureus*, which were used as positive bacterial growth controls. Incubated tubes were inspected daily and declared positive if visible bacterial growth appeared (evidenced via an optical turbidity change compared to the negBRTH). After 7 days of incubation, the negative tubes (no visible turbidity) were discarded, and related samples were declared negative. The positive and negBRTH tubes were then centrifuged at 4750× *g* for 10 min, and the supernatants were collected with sterile syringes. Among positive samples, three of them, obtained from the ovaries of three adult sheep, were randomly selected and used for this study. Small aliquots (1.5 mL) of the three positive FF supernatants, as well as the negBRTH supernatant, were first pH neutralized using a sodium hydroxide 1N solution (Sigma, Milan, Italy, S-2770) or hydrochloric acid 1N solution (Sigma H-9892) to the value of 7.2–7.4, in order to match it with the IVM culture medium conditions, and then filtered using a sterile membrane filter with pore size of 0.22 μm (Merck Millipore, Darmstadt, Germany, SLGS033SB). The bacterial pellets from the sample tubes were resuspended in the residual incubation medium and mixed with the same quantity of sterile 50% glycerol (to obtain the final concentration of the 25% cryoprotectant). The suspension was then aliquoted in two cryovials (1–1.5 mL each), equilibrated at room temperature for 30 min, and frozen at −80 °C for further use. In such a manner, bacterial cell-free FF supernatants (bCFS), named FF-S1, FF-S2, FF-S3, and negBRTH, were prepared, stored at −80 °C, and then used for the IVM-medium supplementation in the study.

### 2.2. 16S rRNA Gene Sequencing

#### 2.2.1. Prokaryotic DNA Extraction

The frozen bacterial stocks (positive samples FF-1, FF-2, and FF-3) were re-cultivated in TgB, and tubes were centrifuged using the same way/conditions as the FFs described above. After discarding the supernatant, bacterial pellets were resuspended and washed in sterile PBS. This content was centrifuged at 2300× *g* for 10 min, the supernatant was discarded, and the obtained bacterial cells were resuspended in 500 µL of sterile PBS. This suspension was further subjected to the amplicon-based 16S rRNA gene sequencing workflow.

The three positive samples were subjected to DNA extraction using the FastDNA™ Spin Kit for Soil (MP Biomedicals, Santa Ana, CA, USA), according to the manufacturer’s instructions. A 40 s bead-beating step at a speed of 6 m/s was executed on the FastPrep Instrument (BIO 101, Carlsbad, CA, USA). The negBRTH and one negative extraction control (nuclease-free water, Ambion DEPC-treated water, Thermo Fisher Scientific, Waltham, MA, USA were also prepared and processed together with the samples to monitor possible contaminations associated with the experimental workflow. The extracted DNA was eluted in a 100 μL volume of sterile water, quantified using a Qubit 1X dsDNA HS Assay (Thermo Fisher Scientific, Waltham, MA, USA), and stored at 4 °C.

#### 2.2.2. 16S rRNA Gene Library Preparation and Sequencing

The V4 hypervariable region of the bacterial 16S rRNA gene was chosen as the target for prokaryotic identification, and the amplicon library was achieved according to established procedures [56]. The V4 region was amplified via polymerase chain reaction (PCR) using 2 ng of DNA extracted from FFs and the universal primer pairs 515F and 806R (underlined nucleotides in the following sequences) containing the transposon Nextera’s sequences (Nextera DNA sample preparation guide, Illumina, San Diego, CA, USA): 515F: 5′-TCGTCGGCAGCGTCAGATGTGTATAAGAGACAG/GTGCCAGCMGCCGCGGTAA-3′, and 806R: 5′-GTCTCGTGGGCTCGGAGATGTGTATAAGAGACAG/GGACTACHVGGGTWTCTAAT-3′. All PCRs were performed in the presence of a negative PCR reaction control (nuclease free water), processed together with the samples. The PCR products were purified using the AMPure XP Beads at a concentration of 0.8 *v*/*v* (Agencourt Bioscience Corporation, Beverly, MA, USA) employing the Hamilton Microlab STAR Liquid Handling System (Hamilton Company, Reno, NV, USA). The obtained amplicon libraries (around 420 bp long) were then quantified using the Qubit 1X dsDNA HS Assay (Thermo Fisher Scientific, Waltham, MA, USA). Equimolar ratios of the purified amplicons were pooled. Considering the insert sizes of the obtained libraries (~450 bp), the paired-end sequencing was performed with V2 chemistry (2 × 250 bp) on the MiSeq platform (Illumina, San Diego, CA, USA). The 20% PhiX sequencing control was added to the pool and co-sequenced [57]. The negative controls were evaluated qualitatively and quantitatively and, as the analyses confirmed the absence of contaminations, these controls were not sequenced.

#### 2.2.3. Bioinformatic Analysis

Raw sequences were analyzed by using the QIIME2 pipeline (version 2019.7; “https://qiime2.org/ accessed on 21 November 2022”) [58] relying on the Amplicon Sequence Variant (ASV) inference and their taxonomic classification. In particular, paired-end (PE) sequences were imported, and the Illumina adapters and PCR primers were removed by using cutadapt [59]. Following the evaluation of sequence quality, the PEs were denoised using the DADA2 plugin [60]. Then, the obtained ASVs were taxonomically annotated by using the feature-classifier (method: classify sklearn) plugin [61] against the Silva database (release 138) [62]. The phylogenetic inference was achieved by using the align-to-tree-mafft-fasttree plugin: a multiple sequence alignment of ASV sequences was obtained by using the MAFFT program [63], and the phylogenetic tree was inferred through the maximum-likelihood procedure implemented in the Fasttree 2 tool [64]. Contaminant ASVs were identified and removed by using the R package Decontam (version 1.18.0) [65]. The R packages phyloseq (version 1.40) [66] and vegan (version 2.6.4) [67] were used to measure alpha and beta diversity. ASV counts were normalized by using rarefaction to perform alpha diversity inference, and then, the Shannon index was calculated. The beta diversity was assessed using the Aitchison distance based on CLR-transformed data [68].

### 2.3. (Targeted) Culturomics

Frozen bacterial pellets were first recovered via re-cultivation in TgB and centrifuged, as previously described. Obtained fresh bacterial pellets were then inoculated on solid cultivation media (agars) using a streak technique. Based on the results obtained via 16S rRNA gene sequencing, multiple cultivation conditions were created to provide a suitable environment for recovery of the detected taxa in the three FFs investigated (FF1, FF2, and FF3). To achieve this, two solid media were used—enriched, non-selective Columbia blood agar base with added sheep blood (Oxoid, Basingstoke, Hampshire, UK), and selective differential MacConkey agar (Biolife, Milano, Italia). Each sample was inoculated on four blood agar and two MacConkey plates (six agar plates per sample, eighteen in total).

Blood plates were incubated in three different atmospheric conditions (aerobic, anaerobic, and an O_2_-reduced/CO_2_-enhanced atmosphere, generated using the candle jar method) and at two different temperatures (30 °C and 37 °C), while MacConkey media only was used aerobically and at the same temperatures as the blood agar plates (30 °C and 37 °C). In this way, we designed six different culture conditions for each specimen (Table 1).

All inoculated plates were incubated for up to 7 days and inspected daily for bacterial growth. Selected colonies (according to their morphological characteristics, including the time of appearance, size, pigmentation, hemolysis on blood agar, and color change of differential medium) were sub-cultivated by using the streak plate method on blood agar to obtain pure cultures. Fresh colonies/strains from pure cultures were ultimately identified via Matrix-Assisted Laser Desorption Ionization–Time-Of-Flight Mass Spectrometry (MALDI-TOF MS, Bruker MALDI Biotyper, Billerica, MA, USA), which profiles bacterial proteins from cultured isolates enabling the discrimination of microorganisms from different genera and species within minutes [69]. The procedure was performed following the manufacturer’s instructions and log scores over 2 were considered as the ‘High Confidence Identification’ of identified bacterial species. Finally, isolated and identified strains were stored at −80 °C for any further needs.

### 2.4. In Vitro Embryo Culture (IVC) of Prepubertal Lamb Oocytes

All the steps of the IVC procedure were performed in 4-well dishes (Nunc Intermed, Roskilde, Denmark).

#### 2.4.1. In Vitro Oocyte Maturation (IVM)

The IVM of PLOs was performed as reported previously [55]. The IVM medium was prepared based on Tissue Culture Medium (TCM)-199 medium with Earle’s salts, buffered with 5.87 mmol/L HEPES and 33.09 mmol/L sodium bicarbonate, and supplemented with 0.1 g/L L-glutamine, 2.27 mmol/L sodium pyruvate, calcium lactate pentahydrate (1.62 mmol/L Ca^2+^, 3.9 mmol/L Lactate), 50 µg/mL gentamicin, 20% (*v*/*v*) FCS, 10 µg/mL of porcine follicle-stimulating hormone (FSH), and luteinizing hormone (LH; Pluset, Calier, Barcelona, Spain) [70], as well as 1 µg/mL 17-beta-estradiol [71]. COCs were placed in 400 μL of IVM culture medium per well, covered with mineral oil, and cultured in vitro for 24 h at 38.5 °C under 5% CO_2_ in air. On the day that the IVM procedure began, aliquots of IVM medium were supplemented with either 20% FCS (used as the control, CTRL), 10% FCS plus 10% negBRTH (used as the vehicle control, vehCTRL), or 10% FCS plus 10% of each FF-S sample. The percentage of FF-S was chosen according to our preliminary data in which this amount of IVM medium replacement was found as non-detrimental for oocyte physiology. For each experiment, at least three replicates were performed, where a replicate was a group of 20–25 COCs cultured for IVM in one plate well of the plate.

#### 2.4.2. In Vitro Fertilization (IVF)

The IVF step was performed in Synthetic Oviductal Fluid (SOF) medium [55] supplemented with 2% estrous sheep serum (OSS) and 1 μg/mL heparin [72,73]. Oocytes with expanded cumuli were partially decumulated and inseminated with frozen–thawed ram sperm cells (1.5 × 10^6^ sperm cells/mL) and cultured for 22 h at 38.5 °C and under a 5% CO_2_ atmosphere. Presumptive zygotes were cultured in SOF with essential and non-essential amino acids (SOF-AAs) and 0.4% bovine serum albumin (BSA) under mineral oil, for 7 days at 38.5 °C in a humidified atmosphere with 5% CO_2_ and 90% N_2_ [55].

### 2.5. Oocyte and Embryo Quality Assessment

#### 2.5.1. Oocyte Staining for Mitochondria and Reactive Oxygen Species (ROS)

Oocytes were washed three times in PBS with 3% BSA and incubated in the same medium with 280 nmol/L MitoTracker Orange CMTMRos (Thermo Fisher Scientific, Waltham, MA, USA) for 30 min at 38.5 °C and a 5% CO_2_ atmosphere [72,74]. Subsequently, oocytes were washed in PBS containing 0.3% BSA and incubated for 15 min at 38.5 °C in 5% CO_2_, in the same medium supplemented with 10 µmol/L 2,7-dichlorodihydrofluorescein diacetate (H_2_DCF-DA) to detect dichlorofluorescein (DCF) and localize intracellular ROS. Finally, oocytes were washed in PBS without BSA and fixed overnight at 4 °C in a 2% paraformaldehyde (PFA) solution in PBS. Negative controls were analyzed after staining with the same dye and an additional 5 min incubation with 5 µmol/L of carbonyl cyanide 3-chlorophenylhydrazone (CCCP; Molecular Probes). Particular attention was applied to avoid photobleaching [55].

#### 2.5.2. Nuclear Chromatin Evaluation of Oocytes and Embryos

To assess nuclear chromatin, after the fixation in 2% PFA solution in PBS, oocytes and embryos were stained with 2.5 µg/mL Hoechst 33,258 in 3:1 (*v*/*v*) glycerol/PBS and examined under an epifluorescence microscope (Nikon Eclipse 600, Nikon Instruments, Firenze, Italy; ×400 magnification) equipped with a B-2A (346 nm excitation/460 nm emission) filter. Oocytes were classified as germinal vesicles (GVs; when showing a round-shape nucleus within decondensed chromatin located in almost a central position), germinal vesicle breakdown (GVBD; when showing a nucleus with fibrillar chromatin located in a marginal position), metaphase I to telophase I (MI to TI), and metaphase II (MII) with the first polar body (PB) extruded [72]. Oocytes showing either multipolar meiotic spindles, irregular chromatin clumps, or the absence of chromatin were considered abnormal [55]. Oocytes with the first PB extruded and one pronucleus were classified as parthenogenetically activated [75]. Embryos were classified according to their number of nuclei and morphology. Micronuclei and lobulated nuclei were considered signs of chromatin damage [73].

#### 2.5.3. Assessment of Ooplasmic Mitochondrial Distribution Pattern

Oocytes at the MII stage were observed at a ×600 magnification in an oil immersion with a Nikon C1/TE2000-U laser scanning confocal microscope (Nikon Instruments, Firenze, Italy). The 543 nm helium/neon laser and the G-2A filter were used to detect the MitoTracker Orange CMTMRos dye (551 nm excitation and 576 nm emission), and the 488 nm argon-ion laser, and the B-2A filter to detect the DCF dye (495 nm excitation and 519 nm emission). Scanning was performed using 25 optical sections from the top to the bottom of each oocyte, with a step size of 0.45 µm to enable a 3D distribution analysis. Mitochondrial distribution patterns were evaluated based on previous studies [72,74] and classified as follows: (1) homogeneous distribution, indicative of a low-energy cytoplasmic state; (2) perinuclear and subplasmalemmal (P/S) distribution, characteristic of healthy cytoplasmic conditions; and (3) irregular distribution, considered abnormal.

#### 2.5.4. Quantification of Bioenergetic/Oxidative Variables and Mitochondria/ROS Colocalization Analysis

In each individual oocyte, MitoTracker and DCF fluorescence intensities and the Manders’ overlap coefficient [76], indicating the extent of mitochondria/ROS colocalization, were measured at the equatorial plane using the EZ-C1 Gold Version 3.70 image analysis software platform for a Nikon C1 confocal microscope (Nikon Instruments, Firenze, Italy). A circular area was drawn to measure only the region including the cell cytoplasm. The fluorescence intensity within the scanned area (512 × 512 pixels) was recorded, and 16-bit images were obtained. The mitochondrial membrane potential (ΔΨ) and intracellular ROS concentrations were recorded as the fluorescence intensity emitted by each probe and expressed as arbitrary densitometric units (ADUs). Colocalization analysis of mitochondria and ROS was performed with the EZ-C1 Gold software (version 3.70). The degree of colocalization was reported with correlation coefficients quantifying the overlap degree between the MitoTracker Orange CMTMRos and DCF fluorescence signals. Mitochondria/ROS colocalization has been reported as a biomarker of healthy oocytes and embryos [55,72,74].

#### 2.5.5. Embryo Development Evaluation

The progression of embryonic development was assessed at day 2 and day 7 after insemination and confirmed by observing nuclear chromatin via epifluorescence microscopy after Hoechst staining [72].

#### 2.5.6. Statistical Analysis

The proportions of oocytes showing different chromatin configurations and mitochondrial distribution patterns and the proportion of cleaved embryos and blastocysts were compared between groups using the Chi Square test. For the oocyte mitochondria and ROS quantification analysis, data (mean ± standard deviation [SD]) were compared using one-way ANOVA, followed by Tukey’s post-hoc multiple comparison test. All analyses were performed using GraphPad software (version 5.03). Differences with *p* < 0.05 were considered statistically significant.

## 3. Results

### 3.1. 16S rRNA Gene Sequencing (Prokaryotic Diversity Analysis)

The three libraries of dual indexed amplicons of 450 bp, targeting the V4 hyper-variable region of the 16S rRNA gene, were successfully sequenced on the MiSeq platform using a 2 × 250 bp PE sequencing strategy. A total of 1,511,853 paired-end (PE) raw reads were generated (average value of 503,951 raw reads/sample). After the trimming, merging, and denoising procedures, about 91% of initial sequences were retained. A total of 244 ASVs were identified and taxonomically assigned. The alpha diversity analysis was investigated by using the Shannon index, which showed a value of 0.58 for sample FF-1, 0.39 for sample FF-2, and 0.014 for sample FF-3 (Supplementary Figure S1a). The beta diversity was measured by applying the Aitchison distance to CLR-transformed data and showed separation between the three sequenced samples (Supplementary Figure S1b). The results of the taxonomic analysis, at the phylum and genus levels, for FF1, FF2, and FF3 are shown in Table 2.

Proteobacteria and Firmicutes represented the common phyla identified in all samples. Proteobacteria resulted in the dominant taxa in FF2 and FF3. Conversely, Firmicutes exhibited the highest relative abundance in FF1 (74.11%), followed by Proteobacteria (25.88%). Regarding the Proteobacteria, the most abundant was *Escherichia-Shigella* in all three samples (25.875% in FF1, 99.507% in FF2, 99.858% in FF3). *Burkholderia-Caballeronia-Paraburkholderia* was detected in FF2 with a relatively low abundance (0.371%), which was still prominently higher than the other abundance values (all below 0.016%). Concerning Firmicutes, the most abundant genus was *Streptococcus* (74.080% in FF1), while the other taxa were presented in extremely low values (less than 0.016%). The phyla Bacteroidota and Actinobacteriota, and belonging genera, were present in all three FFs but with very low abundance (lower than 0.011% in both phyla, Table 2).

### 3.2. (Targeted) Culturomics

We isolated and identified three bacterial species—*Streptococcus infantarius* subsp. *infantarius*, *Escherichia coli*, and *Burkholderia cepacia*, represented by six different strains (Table 3).

From FF-1, S. *infantarius* subsp. *infantarius* and two different strains of non-hemolytic *E. coli*—one lactose ‘+’ (lactose fermenter, concerned as typical strain isolated frequently) and the other lactose ‘−’ (does not ferment lactose, concerned as atypical strains isolated rarely)—were isolated. *E. coli* (lactose ‘+’ and non-hemolytic) and *B. cepacia* were recovered from FF-2, and finally, from FF-3, the identified bacterium was lactose ‘+’ *E. coli*, which differs from the other three isolated *E. coli* strains in the hemolytic activity (α-hemolysis) observed on the blood agar plates.

### 3.3. Effects of In Vitro Oocyte Exposure to FF Microbiota Metabolites During IVM on Oocyte Maturation and Embryo Development

#### Evaluation of Oocyte Maturation and Bioenergetic/Oxidative Status

Oocyte nuclear maturation rates were not affected by replacement of 10% FCS with negBRTH/vehCTRL compared to CTRL. The addition of FF-Ss did not affect oocyte nuclear maturation, indicated as MII rates (Table 4), even if the percentages of oocytes at the GVBD stage (blocked just after resuming meiosis) significantly increased upon the addition of FF-S2 (*p* < 0.05) and that of oocytes showing abnormal chromatin configurations significantly increased upon the addition of FF-S3 (*p* < 0.05).

After culture in the presence of vehCTRL, the percentage of MII oocytes with the P/S pattern did not differ compared to the CTRL (41% vs. 53%; not significant; Table 5).

**Figure 1:**
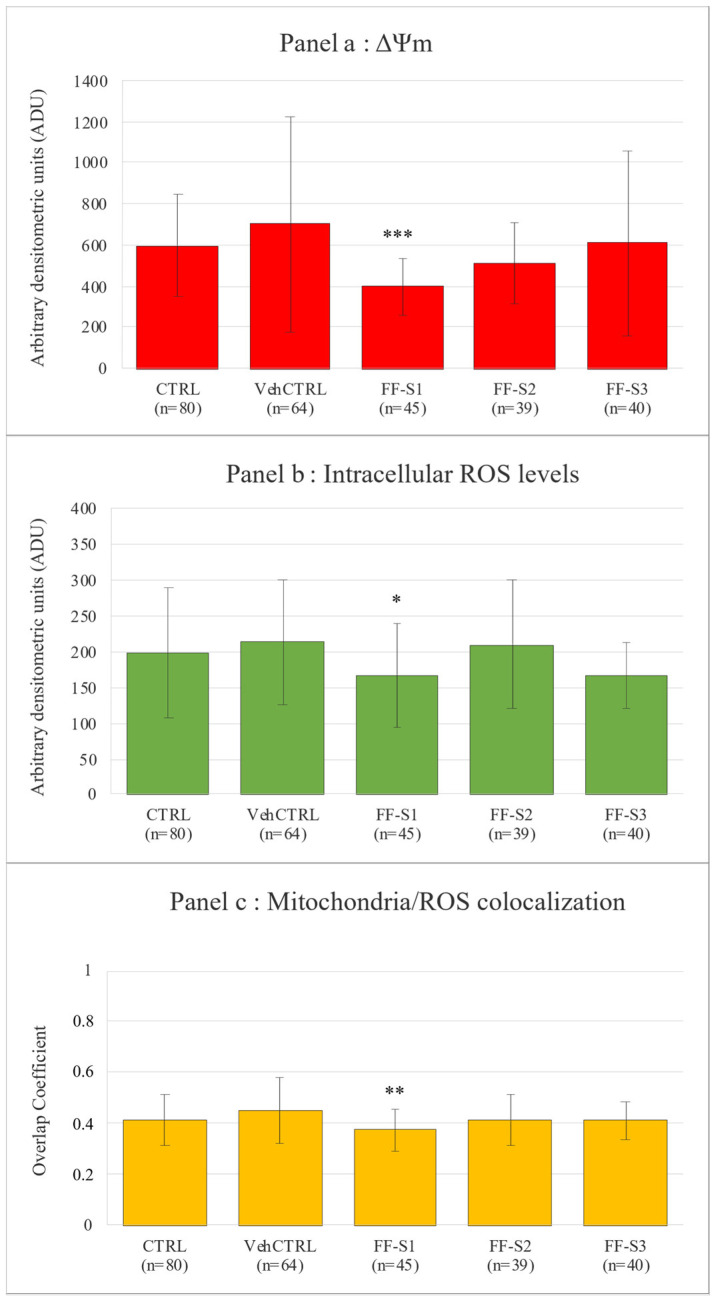
Graphs showing the effects of in vitro exposure to FF microbiota metabolites during IVM on oocyte bioenergetic/oxidative parameters: (**a**) mitochondrial membrane potential, (**b**) intracellular ROS levels, and (**c**) mitochondria/ROS colocalization in PLOs. Mean values ± SD of MitoTracker Orange CMTMRos and DCF intensity of fluorescent labeling in oocytes are expressed as arbitrary densitometry units (ADU). Mitochondria/ROS colocalization is expressed based on the overlap coefficient. Numbers of analyzed oocytes per experimental condition are indicated at the bottom of each bar. One-way ANOVA, Tukey’s post-hoc multiple comparison: FF-exposed versus vehCTRL: * = *p* <0.05; ** = *p* < 0.01; *** = *p* < 0.001.

In addition, bioenergetic/oxidative quantification data did not differ between vehCTRL and CTRL (Figure 1).

**Figure 2:**
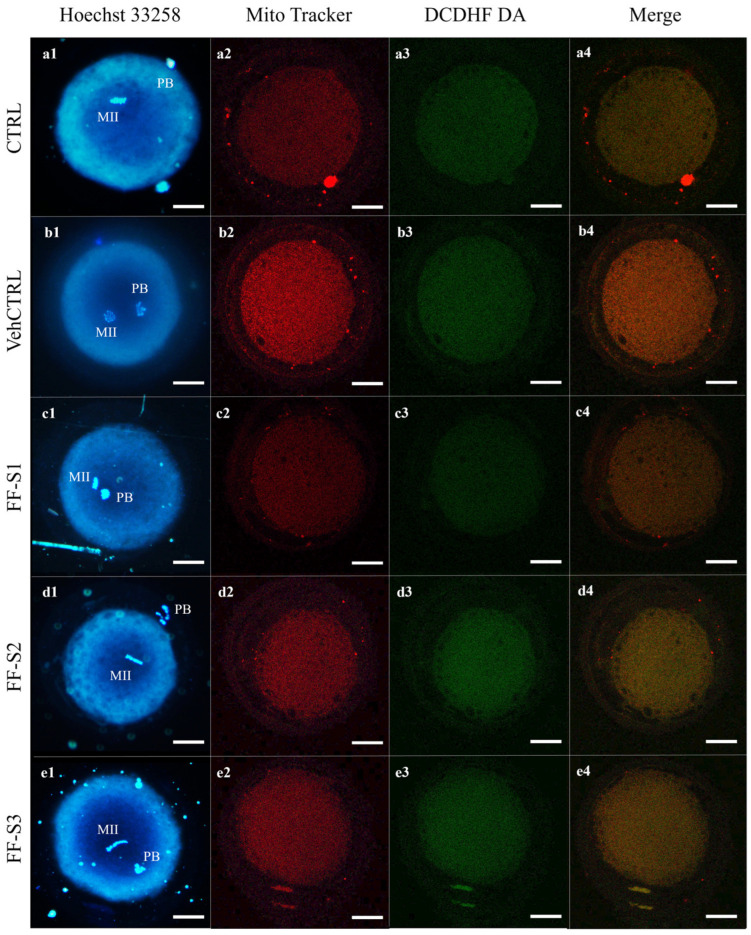
Photomicrographs showing representative images of (**a**) one MII control oocyte and of MII oocytes obtained after IVM in the presence of (**b**) vehCTRL or (**c**) FF-S1, (**d**) FF-S2, and (**e**) FF-S3, respectively. Corresponding epifluorescence images showing the (**1**) nuclear chromatin configuration (Hoechst 33258) and confocal images showing the (**2**) mitochondrial distribution pattern and activity (MitoTracker Orange), (**3**) intracellular ROS localization and levels (H_2_DCF-DA), and (**4**) mitochondria/ROS colocalization (merge). Confocal images were taken at the oocyte equatorial plane. Decreased mitochondrial membrane potential, intracellular ROS levels, and mitochondria/ROS colocalization are visible in FF-S1-exposed oocytes. Scale bars represent 40 μm.

In oocytes with FF-Ss supplementation, the mitochondria distribution pattern was not modified compared with vehCTRL, indicating that these culture conditions allowed this structural and functional aspect of ooplasmic maturity to be preserved (Table 5). The mitochondrial membrane potential, intracellular ROS levels, and mitochondria/ROS colocalization (Figure 1) did not change between CTRL and vehCTRL. In addition, these parameters were not altered upon FF-S2 and FF-S3 addition compared with vehCTRL. Instead, in oocytes matured in the presence of FF-S1, the mitochondria membrane potential (*p* < 0.001), ROS levels (*p* < 0.05), and mitochondria/ROS colocalization (*p* < 0.01) were significantly reduced compared with those cultured with vehCTRL. Figure 2 shows photomicrographs of MII oocytes obtained after IVM (a) in control conditions or (b) in the presence of vehCTRL or (c) FF-S1, (d) FF-S2, and (e) FF-S3, respectively. Decreased mitochondrial membrane potential (c2), intracellular ROS levels (c3), and mitochondria/ROS colocalization (c4) are visible in FF-S1-exposed oocytes.

### 3.4. Evaluation of Embryo Development

Unlike what was observed for oocyte maturation, embryonic development was impaired by the replacement of 10% FCS with negBRTH/vehCTRL compared to the CTRL. Indeed, for both observations on day 2 and day 7, total cleavage rates were significantly lower compared with the CTRL (*p* < 0.05 and *p* < 0.01, respectively). By comparing the different FF supernatants with respect to the vehCTRL it can be noted that, at observation on day 2, supplementation with FF-S3 resulted in a significantly higher percentage of embryos cleaved at the 2–4 cell stage (*p* < 0.01) compared with the vehCTRL. Instead, no effects were noticed after the addition of FF-S1 and FF-S2 (Table 6).

At observation day 7, total embryo cleavage significantly increased after supplementation with FF-S2 (*p* < 0.01) and FF-S3 (*p* < 0.05) compared with vehCTRL. In detail, FF-S2 significantly increased the rate of 4–8-cell stage embryos (*p* < 0.01), and FF-S3 increased the rates of 2–4-cell (*p* < 0.05) and 4–8-cell stage embryos (*p* < 0.05). Instead, in the presence of FF-S1, no effects were observed (Table 6). On day 7, embryo development at the preimplantation stages (morula + blastocyst) was observed after IVM in any examined condition but without statistical significance (Table 6).

## 4. Discussion

To the best of our knowledge, the present study is the first research focused on the adult ovine FF microbiota and the possible effect of its in vitro-produced metabolites, used for IVM supplementation, on PLO competence.

The first step of our research was the enrichment of FF bacteria in liquid medium, motivated by two reasons. The first one was the small amount of FF recovered from slaughtered (not hormonally stimulated) animals along with the fact that FF can be considered a microbiome low-biomass niche. TgB was the liquid medium of choice since it enables the growth of a wide spectra of bacteria according to the nutritional requirements and all types of bacteria regarding the atmospheric conditions (aerobic, anaerobic, facultative anaerobic, and microaerophilic). Besides this ‘recovery expanding’ reason, the second motivation of the enrichment step was the production of secondary metabolites in the medium, later used as bCFS for IVM medium supplementation. In our case, we provided not just the inoculation of all taxa present in the sample but also preserved their genuine abundance providing the metabolic community behavior closest to their natural habitat. A further advantage is that in this way, low-abundance bacteria (<1% of the relative abundance), often neglected in comparison to the high-abundance (dominant) bacteria [77], were included in the experiment as well, maybe influencing metabolic functions of the FF microbiome [78].

In a previous study, it was found that *lactobacillus* bCFS stimulates proliferation of the cells of embryonic stages including bovine embryos, which significantly promote the development of zygotes to the four-cell stage and from the four-cell stage to blastocysts [79]. This was the only study that we found in scientific databases in which a bCFS was introduced in the IVM/IVF/IVC system but, unlike our experiment, the supernatant was added in the last stage of the process (IVC of fertilized oocytes and embryos). According to experiments on the IVM supplementation influence on oocyte developmental competence, including PLOs, available studies can be divided into two groups according to the outcome—positive or negative, as detailed below. Regarding positive outcomes, numerous attempts via several strategies, including IVM media supplementation with different agents but not FF bacteria products and metabolites [40,80,81,82,83], have been performed to enhance the PLO developmental competence. Among them, the study of Tian and colleagues [40] is particularly interesting since they use, as an IVM supplementation, FF from the abattoir-derived FSH-stimulated ovaries of adult sheep. They found significantly increased blastocyst formation rates from lamb oocytes. In their discussion, the authors report that the added FF (as well as estrus serum) is of an unknown composition and that substances that exert positive effects on oocyte competence are still unknown, hypothesizing that diverse concentrations or proportions of some adult FF components (such as fatty acids, estradiol concentration, amounts of many proteins, or antioxidant melatonin) may have a combined effect on oocyte development. However, the FF microbiota presence was not investigated in that study and, although FF supernatants were used to exclude any presence of bacteria in the supplementation, the microbiota metabolites (if any) would still have been introduced into the IVF system, with possible effects on the oocyte maturation process. Also, the possibility of microbiota metabolites introduced by the estrus serum cannot be omitted, since these small molecules could be present in the serum after their production in other body sites (primarily gut). In another study [84], small-extracellular vesicles (sEVs, <200 nm) with a bovine ovarian follicular (granulosa cells and cumulus–oocyte complexes) origin were used for supplementation during oocyte maturation and early embryo development to investigate their role in modulating messenger RNA, microRNA, and global DNA methylation and hydroxy methylation levels in bovine blastocysts. Similarly to the previous example with FF and estrus serum addition, the bovine FF was not tested for microbiota presence, and the applied procedure for EV isolation could not separate potentially present microbial/bacterial extracellular vesicles (bEVs) since their sizes vary from 20 to 200 nm (in Gram-positive membrane vesicles (MVs) [85] and 20 to 400 nm (in Gram-negative outer membrane vesicles, OMVs) [86]. Given the growing body of evidence that bEVs, in addition to mediating microbial inter-species communication, also play an essential role in the inter-kingdom cross-talk interacting with nearby host cells or even traveling through body fluids to distant sites [87], it is plausible to hypothesize that, in experiments where microbiota or bEVs are not explicitly excluded, the outcome may also be influenced by the cargo of these extracellular vesicles. This cargo includes not only small molecules but also macromolecules such as signaling molecules, proteins, DNA, and RNA of bacterial origin [87]. From the available studies showing the negative influence of bacteria products and metabolites on the developmental competence of oocytes, the majority are dedicated to the effects of Gram-negative bacteria lipopolysaccharide (LPS). The addition of LPS to the IVM medium induced bovine oocyte maturation perturbation resulting in their failure to complete meiosis [88], significantly decreased the 1st PB extrusion rate, and delayed cell cycle progression [89] or perturbation of oocyte nuclear maturation and changes in the mitochondrial status [90]. The reduction of meiotic maturation and embryonic development was also noted in porcine oocytes after LPS addition to the IVM medium [91]. In sheep, the response of ovine oocytes to LPS supplementation during IVM, to the best of our knowledge, was reported only in one study [92]. The authors reported the disturbed developmental competence of ovine oocytes by affecting nuclear and cytoplasmic maturation (reduction of oocytes that reached the MII stage, alterations to oocyte mitochondrial membrane potential and ROS levels during maturation, and influences on the transcriptional profile). To the best of our knowledge, the only study that investigated the effects of LPS (or any other bacterial product) exposure on prepubertal ovaries was in vivo and conducted in rats [93], with the conclusion that repeated LPS exposure during the prepubertal period could induce multiple alterations in the steroidogenic machinery in the ovary. Besides LPS, the addition of other bacterial-pathogen-associated molecular patterns (such as lipoteichoic acid, peptidoglycan, and Pam3CSK4) to the IVM medium was evaluated in the study of Bromfield and Sheldon (2011) [88], demonstrating a similar inflammatory response by granulosa cells as in the case of LPS.

To our knowledge, the present study is the first report of bacteria presence in sheep FF. Previous studies have been conducted exclusively on human FF [2,3,4,5,6,7,8,9,10,11,12], with only a few on animal species’ samples such as cattle and pigs. Salary et al. (2020) [16] reported that bovine ovarian FF is not sterile, as they isolated *staphylococci*, hypothesizing that the FF presence of *S. aureus* may partly explain the occurrence of infertility in some dairy cows. In pigs, the recovered taxa were more diverse and belonged to *Pseudomonas*, *Enterobacter*, *E. coli*, *streptococci*, and *Serratia* [13,14,15]. In our study, the 16S rRNA gene sequencing data of the three investigated sheep FF enrichment broths allowed for the detection, based on the SILVA database (release 138), of specific or closely related genera, such as the *Streptococcus*, *Escherichia-Shigella*, and *Burkholderia*-*Caballeronia*-*Paraburkholderia* present in relatively higher abundances compared to the others. The FF samples exhibit poor biodiversity, as supported by the low alpha diversity values, with certain taxa notably appearing in very low abundances (Table 2). This data could be associated with the abundant presence of specific taxa, potentially influenced by cell culture conditions, which might also be favored during the sequencing run.

The 16S rRNA gene sequencing data from our enriched FF samples proved highly beneficial for our subsequent culturomics approach. This enabled us to employ various solid media and incubation conditions suitable for the bacterial genera identified, facilitating a targeted approach to isolate specific bacterial species. We avoid the complicated, costly, and time-consuming isolation (typical for a classical culturomics approach) and narrow the creation of isolation conditions to six (Table 1). Additionally, 16S rRNA gene sequencing provided us with relative abundance values of various bacterial genera within the broth. These assignments allowed us to discriminate between high- and low-abundant bacteria, which is crucial as the bacterial load could be helpful for an assessment of the obtained results [9]. Through culturomics, we were able to identify three bacterial species—*E. coli*, *Streptococcus infantarius* subsp. *infantarius* and *Burkholderia cepacia*. When comparing the taxa detected or isolated in both animals and humans in previous studies with our data, we observe that *E. coli* is reported in both, as well as streptococci as a genus. But, to the best of our knowledge, the species *Streptococcus infantarius* subsp. *infantarius* and *Burkholderia cepacia* are not previously reported in any of the mentioned studies. Therefore, we can conclude that these species (and *Burkholderia* as genus) are, for the first, time reported in FF, either animal or human.

As we can see from the results, various bacterial 16S rRNAs were detected via the sequencing procedure. This finding raises the question of their origin since one of the main doubts in FF microbiota investigations, particularly in humans, is “are they genuine (FF colonizers) or contamination occurred during the sampling procedure” [8]. For the cultivable microbiota, isolated bacteria are, almost certainly, not contamination since, beside positive samples, actually even more negative FF samples were found. Therefore, the applied procedure of reproductive tract/ovary decontamination and the use of a thin 27-gauge needle minimized the introduction of bacteria from the ovary surface into the FF, at least for the quantities that will allow abundant multiplication and subsequent isolation. On the other hand, for the detected nucleic acid presence, we cannot exclude the possibility of the introduction of a minor number of bacterial cells, remaining after the decontamination procedure, of which quantities are insufficient for abundant multiplication and isolation but will allow for positive molecular detection (at a low relative abundance as we can see from Table 2). Additionally, we should take into account the origin of the FF as a mixture of oocyte/granulosa cell secretive activities and, more importantly, for contamination issues elaborated on for blood plasma components. Although the existence of the blood microbiome was and still is a debatable issue (reviewed in [94]), the development of molecular techniques allowed for the detection of bacterial 16S ribosomal RNA genes in the blood of healthy individuals [95] suggesting very likely commensal microbes’ translocation to the bloodstream from other body sites (e.g., the gut or the skin–oral–gut axis, summarized in [94]). This could provide an explanation for the many different bacterial nucleic acid presences in FF samples that are actually blood plasma filtrates.

Besides upgrading the knowledge on the ovarian FF microbiota by analyzing ovine FFs, the other goal was to evaluate whether the metabolites of the adult FF microbiota could support PLO maturation and to evaluate whether differences between FF metabolites could have a different impact related to the different bacterial taxa present in the investigated FFs.

The IVM experiment was designed to evaluate the short-term effects of FF microbiota metabolites on oocyte nuclear and cytoplasmic maturation, with both aspects expressing oocyte ability to undergo normal fertilization and development [96]. It was observed that FF-S2 and FF-S3 did not affect cytoplasmic maturation compared with the vehCTRL, whereas FF-S1 supplementation reduced the values of all three bioenergetic/oxidative quantitative parameters. These results could be explained considering that the presence of different bacteria in FFs probably resulted in the production of various metabolites with different effects on the COC maturation potential. A bioenergetic analysis of oocytes can help in elucidating the effects of metabolite diversity [55,73]. It can be hypothesized that metabolites from FF-S1 may have activated apoptotic pathways and compromised cell viability in a subset of matured oocytes, while metabolites from FF-S2 and FF-S3, although not enhancing ooplasmic bioenergetics, may have improved the molecular or biochemical aspects of ooplasmic maturation, effects that could become evident only during subsequent embryo development as a long-term impact.

Due to these reasons, the IVC experiment was designed to assess whether FF-S supplementation during IVM could have influenced embryo development, as a long-term effect on the oocyte quality. In fact, it is well known that events occurring during oocyte maturation have relevance on oocyte developmental competence [96,97,98]. In the present study, the impact of FF-S1 metabolites on oocytes did not affect embryo development despite the lowering of energy levels. It can be assumed that persistent oocyte energy levels were still sufficient to ensure embryonic development. On the other hand, the enhancing effects of FF-S2 and FF-S3 metabolites were observed at day 2 and 7. These effects were mainly referred to the early stages, suggesting that metabolites produced by bacteria present in FFs from large developing follicles might not be sufficient to sustain late developmental stages. Future studies using FF-Ss obtained from preovulatory follicles microbiota could possibly improve the results. An alternative hypothesis is that the difference between the FF-Ss of large developing follicles and those of preovulatory follicles may not depend on the type of bacteria present but in the hormonal environment of the preovulatory phase, possibly acting on bacterial metabolism. Further species-specific studies should be conducted to evaluate whether bacterial growth in FFs can be modified by the preovulatory hormone milieu.

Our observations, although obtained using in vitro cell models, are in line with those reported in previous studies in vivo in humans, where the positive, negative and/or neutral effects of the human FF microbiota presence were reported in different steps of assisted reproductive procedures [2,3,4,5,6,7,8,9,10,11,12].

Since in all the studies these bacteria were part of the complex bacterial communities, it is plausible to hypothesize that not just bacteria presence but also inter-species (bacteria–bacteria) influences could be important for the inter-kingdom (bacteria–host) communication. All this variability, as we mentioned before, is noted in human samples, oocytes, and embryos, where the maturation is performed in vivo. However, short-term vs. long-term variability in oocytes and embryos responses was also reported in the studies of animal (bovine) in vitro oocyte maturation where the oocytes were exposed to the purified LPS [99] or Gram-negative endotoxin of *E. coli* and Gram-positive toxin of *S. aureus* [100]. In the first study, COCs were challenged with LPS during IVM, with decreased nuclear maturation but no effect on early embryogenesis (cleavage and blastocyst rates) [99]. In the second, bovine FF was treated with Gram-positive or Gram-negative bacteria and then aspirated and used as maturation medium for in vitro embryo production with a variable impact on oocyte nuclear and cytoplasmic maturation and then oocyte developmental competence [100]. These findings also support our study outcomes of diversity in the short- and long-term effects of in vitro-matured oocytes and consecutive embryogenesis. However, more studies are certainly necessary to establish correlations between FF microbiota/metabolites and their effects on oocyte and embryo development.

## 5. Conclusions

For the first time, we isolated bacteria from sheep FF, with the first report (either in animal or human FFs) of two bacterial species—*Streptococcus infantarius* subsp. *infantarius* and *Burkholderia cepacia* (and *Burkholderia* as a genus, as well). The applied combination of bacteriological and molecular techniques could be used as an efficient tool for the isolation of cultivable microbiota from low-biomass niches/samples, facilitating the labor-intensive characterization of a standard culturomics approach. The bacterial metabolites introduced into the IVM/IVF/IVC systems confirmed the previous observations that short-term bacterial effects on in vitro-matured oocytes not always resemble the long-term impact on embryo development. The reproductive impact of bacterial products could vary not just between different bacterial genera but also in the same bacterial species (positive effect of *E. coli* products on early embryo development). IVM/IVF/IVC experiments with animal FF microbiota could provide information for their contribution in both, animal and human reproductive biology, predicting future research targets including the possible discovery of novel therapeutics in the form of secondary metabolites.

## Figures and Tables

**Table 1:** Designed incubation conditions for each FF sample.

Condition #	Medium	Atmosphere	Temperature
1	Blood agar	Aerobically	30 °C
2	Blood agar	Aerobically	37 °C
3	Blood agar	Anaerobically	30 °C
4	Blood agar	Candle jar (O_2_ reduced/ CO_2_ enhanced atmosphere)	37 °C
5	MacConkey agar	Aerobically	30 °C
6	MacConkey agar	Aerobically	37 °C

**Table 2:** 16S rRNA gene relative abundances (%) of FF samples at phylum and genus levels.

	FF1	FF2	FF3
Phylum: Proteobacteria	25.88	99.90	99.89
Genus:			
*Escherichia-Shigella*	25.875	99.507	99.858
*Burkholderia-Caballeronia-Paraburkholderia*	0.000	0.371	0.016
*Oligella*	0.000	0.013	0.003
*Pseudomonas*	0.003	0.004	0.001
*Achromobacter*	0.000	0.002	0.003
*Uncultured*	0.000	0.001	0.003
*Enhydrobacter*	0.000	0.000	0.000
*Brevundimonas*	0.001	0.000	0.000
*Uncultured*	0.000	0.001	0.000
*Methylobacterium-Methylorubrum*	0.000	0.000	0.001
*Pseudoxanthomonas*	0.000	0.000	0.000
	**FF1**	**FF2**	**FF3**
Phylum: Firmicutes	74.114	0.069	0.081
Genus			
*Streptococcus*	74.080	0.033	0.023
*Enterococcus*	0.019	0.009	0.013
*Staphylococcus*	0.015	0.023	0.035
*Lactobacillus*	0.000	0.001	0.002
*Faecalibacterium*	0.000	0.0004	0.003
*Blautia*	0.000	0.000	0.002
*Fusicatenibacter*	0.000	0.001	0.000
*Roseburia*	0.000	0.000	0.002
*Aerococcus*	0.000	0.001	0.000
*Christensenellaceae_R-7_group*	0.000	0.000	0.000
*Coprococcus*	0.000	0.000	0.0005
*Leuconostoc*	0.000	0.000	0.001
	**FF1**	**FF2**	**FF3**
Phylum			
Bacteroidota	0.000	0.004	0.011
Genus			
*Bacteroides*	0.000	0.002	0.003
*Cloacibacterium*	0.000	0.000	0.006
*Prevotella*	0.000	0.000	0.001
*Empedobacter*	0.000	0.002	0.000
*Alistipes*	0.000	0.0004	0.000
*Parabacteroides*	0.000	0.000	0.000
*Chryseobacterium*	0.000	0.000	0.001
	**FF1**	**FF2**	**FF3**
Phylum			
Actinobacteriota	0.006	0.020	0.016
Genus			
*Corynebacterium*	0.000	0.003	0.004
*Dietzia*	0.000	0.002	0.000
*Cutibacterium*	0.004	0.011	0.008
*Kocuria*	0.002	0.001	0.000
*Georgenia*	0.000	0.000	0.004
*Lawsonella*	0.000	0.001	0.000
*Nocardioides*	0.000	0.001	0.000

**Table 3:** Isolated and identified bacterial strains in three follicular fluids.

FFs	Bacterial Species (Strain)	MALDI-TOF MS ID Log Score
FF-1	*Streptococcus infantarius* subsp. *Infantarius*	2.19
	*Escherichia coli* (lactose ‘+’, non-hemolytic)	2.12
	*Escherichia coli* (lactose ‘−’, non-hemolytic)	2.48
FF-2	*Escherichia coli* (lactose ‘+’, non-hemolytic)	2.26
	*Burkholderia cepacia*	2.19
FF-3	*Escherichia coli* (lactose ‘+’, α-hemolytic)	2.44

**Table 4:** Effects of in vitro exposure to FF microbiota metabolites during IVM on oocyte nuclear maturation.

Well Supplement	N° of Evaluated Oocytes (Replicates)	Nuclear Chromatin Configurations Number (%)					
		GV	GVBD	MI-TI	MII	Abnormal	Activated
CTRL	120 (5)	12 (10)	10 (8)	7 (6)	80 (67)	7 (6)	4 (3)
vehCTRL	93 (4)	8 (9)	7 (8) ^a^	6 (6)	64 (69)	6 (6) ^a^	2 (2)
FF-S1	70 (3)	8 (11)	4 (6)	4 (6)	45 (64)	9 (13)	0 (0)
FF-S2	72 (3)	12 (17)	13 (18) ^b^	4 (6)	39 (54)	3 (4)	1 (1)
FF-S3	72 (3)	5 (7)	8 (11)	7 (10)	40 (55)	12 (17) ^b^	0 (0)

Legend: GV = germinal vesicle; GVBD = germinal vesicle breakdown; MI-TI (metaphase I—telophase I), MII (metaphase II). For each experimental condition, 3 to 5 replicates were performed. Chi square test: comparisons of CTRL versus vehCTRL—not significant; in the same column, comparisons FF-exposed versus vehCTRL—a, b = *p* < 0.05.

**Table 5:** Effects of in vitro exposure to FF microbiota metabolites during IVM on oocyte mitochondria pattern.

Well Supplement	N° of Analyzed MII Oocytes	Mitochondria Distribution Pattern Number (%)		
		Perinuclear and Subplasmalemmal	Small Aggregates	Abnormal
CTRL	80	42 (53)	37 (46)	1 (1)
vehCTRL	64	26 (41)	38 (59)	0 (0)
FF-S1	45	20 (44)	24 (53)	1 (2)
FF-S2	39	16 (41)	23 (59)	0 (0)
FF-S3	40	19 (48)	20 (50)	1 (2)

Note: Chi square test comparisons CTRL versus vehCTRL—not significant.

**Table 6:** Effects of in vitro oocyte exposure to FF microbiota metabolites during IVM on embryo development.

Well Supple-ment (Replicates)	N° of Insemi-nated Oocytes	Embryo Development to N (%)/Inseminated									
		Day 2				Day 7					
		2–4 Cell	4–8 Cell	8–16 Cell	Total Cleaved/ Insemi-nated	2–4 Cell	4–8 Cell	8–16 Cell	M + B	Total Cleaved/ Insemi-nated	M + B/Cleaved
CTRL (7)	168	19 (11)	22 (13)	12 (7)	53/168 (32) ^x^	19 (11)	17 (10)	20 (12)	4 (2)	60/168 (36) ^z^	4/60 (7)
vehCTRL (7)	161	14 (9) ^a^	15 (9)	5 (3)	34/161 (21) ^y^	9 (6) ^a^	11 (7) ^a^	12 (7)	4 (2)	36/161 (22) ^a w^	4/36 (11)
FF-S1 (4)	89	8 (9)	13 (15)	1 (1)	22/89 (25)	9 (10)	12 (13)	5 (6)	3 (3)	29/89 (33)	3/29 (10)
FF-S2 (6)	133	20 (15)	17 (13)	1 (1)	38/133 (29)	12 (9)	22 (17) ^c^	9 (7)	5 (4)	48/133 (36) ^c^	5/48 (10)
FF-S3 (5)	91	21 (23) ^c^	7 (8)	0 (0)	28/91 (31)	13 (14) ^b^	15 (16) ^b^	4 (4)	2 (2)	34/91 (37) ^b^	2/34 (6)

Legend: M = morula; B = blastocyst. For each experimental condition, 4 to 7 replicates were performed, with each replicate consisting of 20–25 COCs cultured for IVM and then inseminated and cultured for IVC. Chi square test: in the same column, comparisons of CTRL versus vehCTRL—x, y = *p* < 0.05; z, w = *p* < 0.01; in the same column, comparisons of FF-exposed versus vehCTRL a, b = *p* < 0.05; a, c = *p* < 0.01.

## Data Availability

The data presented in this study are available on request from the corresponding author.

## Supplementary Materials

The following supporting information can be downloaded at: https://www.mdpi.com/article/10.3390/ani15131951/s1, Figure S1: Alpha and beta diversity analysis of the sequenced FF samples. (a) Shannon index plot for alpha diversity analysis performed on normalized ASV counts for the three sequenced samples. (b) PCoA plot of beta diversity based on Aitchison distance. Each point represents the single sequenced sample.
