# Supplementary material for: Taxonomic Profile of Cultivable Microbiota from Adult Sheep Follicular Fluid and Its Effects on In Vitro Development of Prepubertal Lamb Oocytes
Source: Animals (Basel). 2025 Jul 2;15(13):1951. doi: 10.3390/ani15131951 (PMC12249267; doi:10.3390/ani15131951)
Supplement: Supplementary file 1 [file animals-15-01951-s001.zip › animals-3682036-supplementary.pdf]

Supplementary Figure S1: Alpha and beta diversity analysis of the sequenced FF samples. (a) Shannon index plot for alpha diversity analysis performed on normalized ASV counts for the three sequenced samples. **(b)** PCoA plot of beta diversity based on Aitchison distance. Each point represents the single sequenced sample.

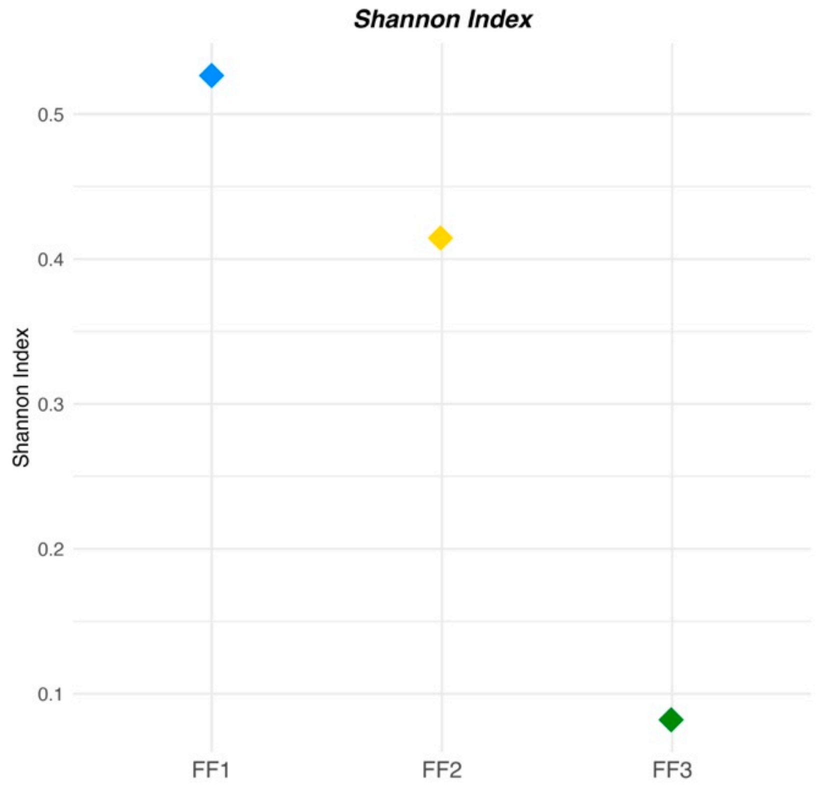

(a)

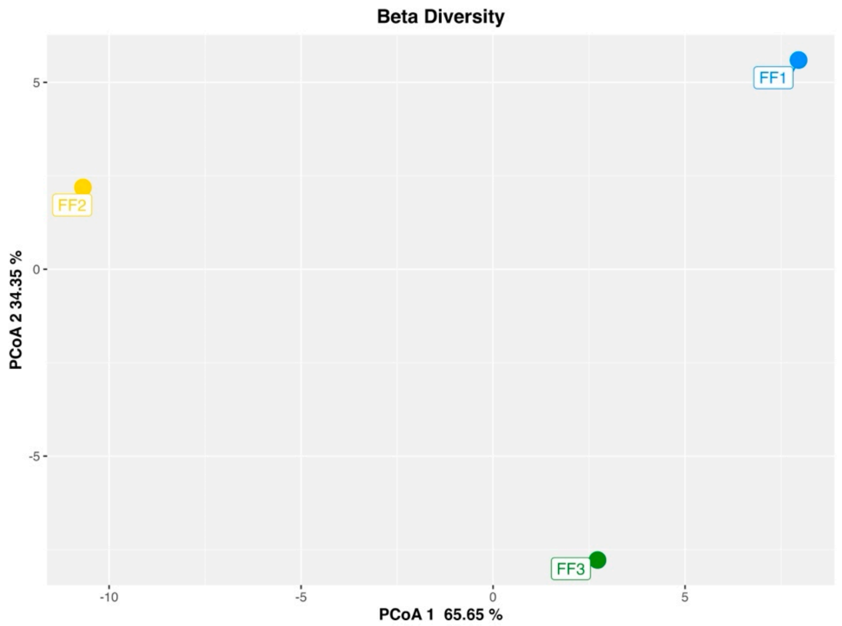

(b)
